# An Update on Physical Activity Research among Children in Hong Kong: A Scoping Review

**DOI:** 10.3390/ijerph17228521

**Published:** 2020-11-17

**Authors:** Chun-Qing Zhang, Pak-Kwong Chung, Shi-Shi Cheng, Vincent Wing-Chun Yeung, Ru Zhang, Sam Liu, Ryan E. Rhodes

**Affiliations:** 1 Department of Sport, Physical Education and Health, Hong Kong Baptist University, Hong Kong 999077, China; shischeng2@hkbu.edu.hk (S.-S.C.); wcvyeung@hkbu.edu.hk (V.W.-C.Y.); 2 Department of Psychology, Sun Yat-Sen University, Guangzhou 510006, China; 3 School of Physical Education & Sports Science, South China Normal University, Guangzhou 510006, China; ruzhang@m.scnu.edu.cn; 4 School of Exercise Science, Physical and Health Education, University of Victoria, Victoria, BC V8P 5C2, Canada; samliu@uvic.ca (S.L.); rhodes@uvic.ca (R.E.R.)

**Keywords:** physical activity, exercise, child, youth, research synthesis, review

## Abstract

Similar to their Western counterparts, children in Hong Kong generally fail to reach the recommended levels of physical activity (PA). As an ultra-dense metropolis, Hong Kong is different from most Western cities. It is therefore important to update and appraise previous PA research in order to inform future PA promotion for Hong Kong children. Using a scoping review, the current study aimed to evaluate PA research among preschool and school-aged children in Hong Kong aged 3–12 years old who are at a critical development stage. Literature was searched from four English databases: Medline via EBSCOhost, SPORTDiscus, ERIC and PsycINFO via ProQuest; and three Chinese databases: CNKI, CQVIP and WAN-FANG. PA research among Hong Kong children published from 1 January 1997 to the searching date, 31 March 2020 was included. A total of 63 studies were identified, with the majority of studies focused on school-aged children as compared to preschoolers, adopted a cross-sectional design, using self-reported PA measures, and with small to medium sample sizes. We classified eligible studies into five main categories: (a) Health benefits of PA (*k* = 12). Consistent evidence on the health benefits of skeletal and cardiovascular capacity, quality of life, cognitive function, and sleep quality was revealed. However, inconsistent evidence was found on the benefits of weight-related indicators and academic performance. (b) Patterns of PA (*k* = 12). There is a general pattern of low levels of PA among Hong Kong children, in particular girls and children with special educational needs. (c) Measures of PA and related constructs (*k* = 11). The Chinese versions of self-reported measures of PA, PA-related social environment, and PA-related psychological constructs showed acceptable reliabilities and validities. (d) Correlates of PA (*k* = 18). The correlates of PA include physical environment, social environment, physical factors, psychological factors, and multiple correlates, which is in line with the social-ecological model. (e) Interventions for promoting PA (*k* = 10). PA interventions among Hong Kong children were conducted for healthy children, children with special educational needs, and children with cancer. Overall, there is a growing volume of PA research among children in Hong Kong in the recent decade. Yet, there is a lack of high-quality research for measuring, understanding, and promoting PA among Hong Kong children. It is highly recommended that future PA research among children should pay more attention on the preschoolers, adopting robust research design (e.g., randomized controlled trials), recruiting large and representative sample, and collecting device-assessed data.

## 1. Introduction

During the past two decades, there has been a growing trend worldwide that children have become physically inactive [1,2,3]. Hong Kong, one of the most high-income and population dense regions in the world, faces the same problem. Generally, Hong Kong children lack sufficient physical activity (PA) across all modes. Recently, the Hong Kong 2018 report card summarized the PA indicators for children and youth indicating that the overall PA for Hong Kong children and youth was C- grade (i.e., 40–46% of children and youth meet the PA guidelines of an average of 60 min moderate-to-vigorous PA per day), as compared to the *D* grade (i.e., 27–33% of children and youth meet the PA guidelines) in the Hong Kong 2016 report card [4]. Progress was made, but the percentage of PA active population among Hong Kong children was still not high enough. Very recently, the results of Hong Kong’s 2019 report card on PA for children and youth with special educational needs indicated that the overall PA indicator for the special population was *F* grade (i.e., less than 20% of children and youth meet the PA guidelines of an average of 60 min moderate-to-vigorous PA per day), indicating this population had extremely low PA levels [5]. Nonetheless, it seems that there is lack of PA research for children and youth in Hong Kong with limited data sources available at the 2016 and 2018 report cards for healthy children and youth as well as the 2019 report card for children and youth with special educational needs.

The health benefits of participating in regular PA for schoolchildren have been well-documented [6,7,8]. High levels of PA in children and adolescents provide health benefits such as the reduction of risk factors of cardiovascular disease, and adiposity, whereas PA imparts an increase in bone health [6]. It is suggested that at least moderate intensity PA can lead to substantial health benefits [7,8], with dose-response relations analysis indicating that more PA confers higher levels of health benefits [7]. Given the health benefits of regular PA among children, it is urgent and important to promote PA among children with empirical evidence. As such, there is a need to systematically understand the determinants of PA among children in order to develop effective PA promotion interventions for children and youth [9].

Over the past decade, promoting PA among children with the development of the appropriate polices has been well recognized worldwide [10,11]. Notably, the effectiveness of PA promotion programs can be increased by following the principles of policy development more closely [12]. In Hong Kong, the government has always valued the importance of promoting PA in the community. For example, the Department of Health of Hong Kong government [13] released the “Action Plan to Promote Healthy Diet and Physical Activity Participation in Hong Kong” in order to overcome the physical inactivity problems in Hong Kong. In 2018, the Department of Health of Hong Kong government [14] formulated and endorsed a strategy and action plan “Towards 2025: Strategy and Action Plan to Prevent and Control Non-communicable Diseases in Hong Kong” aiming to prevent and control non-communicable diseases, including physical inactivity. With these policy-wise plans, tailored strategies that were built on the evidence of local population is required.

Culturally-sensitive and context-specific PA promotion programs are key for successful PA promotion [15]. This is because Hong Kong is a unique context as the region contains intersects of traditions and practices based on its history, which heavily influenced by both Eastern and Western cultures. Geographically, Hong Kong is an ultra-dense metropolis with urban environments for PA different from most Western cities. This highlights the importance of summarizing the existing evidence of PA research among children in Hong Kong. In a previous study, He and colleagues [16] reviewed literature of PA research among children and adolescents in Hong Kong published between 1987 and 2012 and divided the PA research into five categories: health benefits of PA, participation in PA, assessment of PA, correlates of PA, and interventions to promote PA. In the review of He and colleagues [16], majority of the studies focused on the aspects of health benefits of PA, participation in PA, and correlates of PA. Despite the valuable information provided in this review, there were noteworthy limitations: (a) this review only focused on healthy youth from four to 18 years old, excluded the PA research for youth with special educational needs or diseases, and (b) PA research among children and adolescents were summarized together in this review, although the patterns, correlates, and interventions of PA on children aged 3–12 years old and adolescents aged 12–17 years old are different [17]. In order to provide population specific suggestions, a separate review for children and adolescents is necessary. Furthermore, seven years have passed since the search date of the review (i.e., December 2012) [16] and PA research on Hong Kong children has likely proliferated. It is therefore necessary to continue to update this literature with a focus on the population of children.

Overall, it is timely and important to regularly take stock of the research literature on PA research among children in Hong Kong in order to inform policymaking and developing effective intervention programs for promoting PA among Hong Kong children. Therefore, the current study aimed to conduct a scoping review to summarize the PA research among children in Hong Kong aged 3–12 years old, which covers both the preschool-aged children (3–5 years old) and school-aged children (6–12 years old) that are the critical period to cultivate the PA habits which can transit to their adolescents. It should be noted that a systematic review is not feasible due to the broad aims rather than specific aims with a definitive research question.

## 2. Methods

In this scoping review, we reviewed the PA research among children in Hong Kong that were published from January 1, 1997 to March 31, 2020, which is the date conducting the literature searching. This is due to the fact that no PA studies among Hong Kong children had been published before the year of 1997 [16]. We searched relevant studies four English databases, including: MEDLINE (EBSCOhost), SPORTDiscus, and ERIC and PsycINFO (ProQuest), and three Chinese databases, including: CNKI, CQVIP and WAN-FANG. We used the keywords “physical activity OR physical training OR physical fitness OR exercise “, “children OR pre-school children OR child “, and “Hong Kong”. We translated the English keywords into corresponding Chinese words for use in the Chinese databases.

We developed the inclusion and exclusion criteria for screening the eligible studies. Inclusion criteria were: (a) participants have to be children in Hong Kong; (b) children’s mean age should fall within the range from three to 12 years old; and (c) PA should be the of the major dependent or independent variables. It should be noted that all types of PA (e.g., PE lessons, active travel, and sport participation) were considered for inclusion. Exclusion criteria were: (a) review articles and protocols without data; (b) full text conference abstracts that are unavailable; and (c) children in Hong Kong are only part of the sample but not the whole sample.

Based on the aforementioned inclusion and exclusion criteria, two reviewers (VWY and SSC) independently screened the titles and abstracts of studies after the removal of duplicated records. Then, the two reviewers screened the full-text articles, and removed articles that failed to meet the inclusion criteria. For hand searching, the two reviewers further searched reference lists of eligible articles and the prior review [16], and articles that cited the eligible articles using the search engine of Google Scholar. Group discussions were conducted to resolve the discrepancies among the lead author (CQZ) and the other two reviewers (VWY and SSC).

We followed the classification of PA studies by He and colleagues [16], and divided the existing PA research among Hong Kong children into five similar categories: (a) health benefits of PA, (b) patterns of PA, (c) measures of PA and related constructs, (d) correlates of PA, and (e) interventions for promoting PA. The classification decision was made by appraising the key study information reported and the main purposes of the studies. The three reviewers (CQZ, VWY, and SSC) completed the classification. In the event of conflicting opinions, resolution was achieved by consensus between three reviewers. The PRISMA extension for scoping reviews (PRISMA-ScR) [18] was followed for reporting the findings. Given the nature of scoping review, risk of bias assessment is also not applicable.

## 3. Results

**Figure 1:**
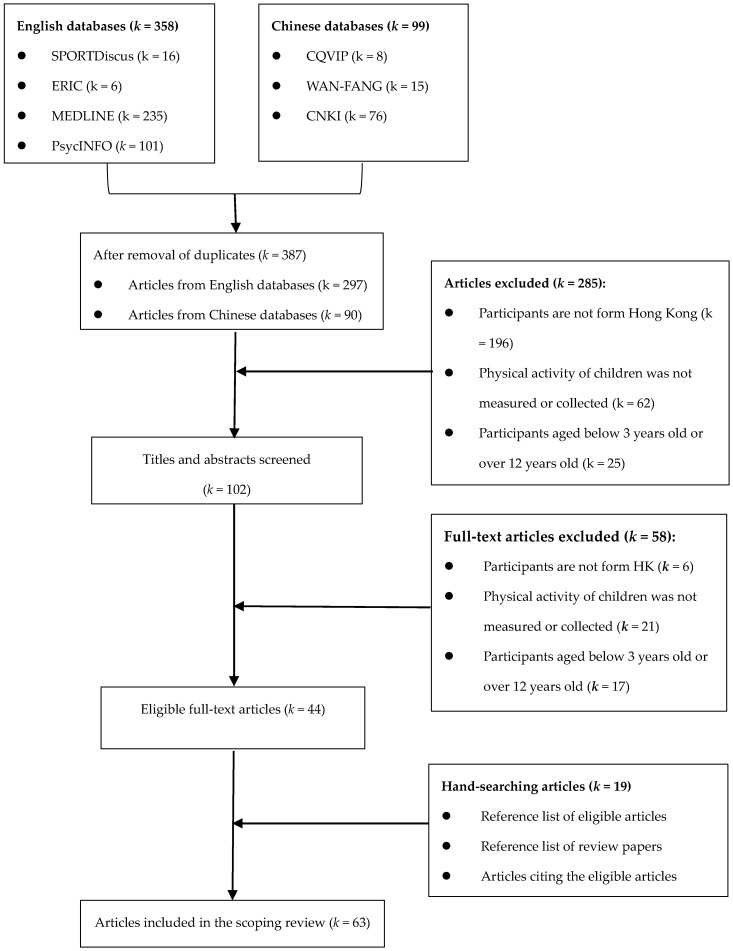
Article Screening Flowchart.

The initial search yielded a total of *k* = 457 articles, with *k* = 358 articles from English databases and *k* = 99 from Chinese databases. Following the removal of duplicates, full-texts of *k* = 102 articles were screened by the two reviewers (VWY and SSC) and the interrater reliability was ICC = 0.8446 (*p* < 0.01). A total of *k* = 44 articles remained and we found *n* = 19 articles from manual searching. Accordingly, there is a total of *k* = 63 articles included for the scoping review. We described the searching process and outcome in Figure 1.

### 3.1. Study Characteristics

As indicated in Table 1, the majority of PA research on children in Hong Kong has been conducted after 2011, with *k* = 20 articles published at the period of 2011–2015 (31.7%) and *k* = 25 articles published at the period of 2016–2020 (39.7%). As for the age range of participants, a majority of the studies focused on the school-aged children 6–12 years old (81.0%), with a relatively small percentage of studies focused on preschool children aged 3–5 years old (14.3%), and even less studies covering both the preschool and school-aged children (4.8%).

Regarding the sample sizes of the eligible studies, there are *k* = 22 studies with a relatively small sample size of 100 and below participants (36.1%), *n* = 24 studies with medium sample size of 101–500 participants (39.3%), eight studies with medium to large sample size of 501 to 1000 participants (13.1%), and seven studies with large sample size of 1001 and above participants (11.5%). There were 44 studies focusing on healthy children (69.8%), and 13 studies targeting children with special educational needs (28.6%), and six studies on children with a disease (9.6%). For the types of special educational needs, there five studies targeting children with coordination disorder (38.5%), three studies targeting cerebral palsy (30.8%), two studies for multiple disabilities (15.4%), one study for autism spectrum (7.7%), and one study for intellectual disorder (7.7%). Regarding the study design, the majority of studies adopted a cross-sectional design (*k* = 40; 63.5%), with eight longitudinal studies (12.7%), and three qualitative studies (4.7%). There were 10 studies (15.9%) that employed randomized controlled trials (RCT) and two studies that used the quasi-experimental design (3.2%). For the measures of PA, the majority of the studies used single type of self-report measures (*k* = 44, 69.8%), with 11 studies (17.5%) used single type of device measures (e.g., accelerometers and/or pedometers), six studies used the combined type of self-report measures and devices (9.5%), and two studies used the single type of objective observational measures (3.2%).

### 3.2. Main Findings

Similar to the previous review [11], all the eligible studies (*n* = 63) were classified into five main categories: health benefits of PA (*k* = 12), patterns of PA (*k* = 12), measures of PA and related constructs (*k* = 11), correlates of PA (*k* = 18), and interventions for promoting PA (*k* = 10). It should be noted that the description of the main findings in this scoping review intend to be primarily descriptive and the evaluative orientation is reserved for the discussion section.

#### 3.2.1. Health Benefits of PA

Twelve studies explored the health benefits of PA in Hong Kong children (see Table 2). The majority of studies (*k* = 10) used a cross-sectional design, with only two studies that used the RCT design. Evidence on the health benefits of PA on skeletal, cardiovascular capacity, quality of life, sleep quality, and cognitive function were clear and consistent. However, mixed findings were revealed on the health benefits of PA on weight-related indicators and academic performance.

The health benefits of PA on skeletal and cardiovascular capacity of Hong Kong children were preliminarily established. For example, limited PA participation intensity was found associated with delayed skeletal development of Hong Kong children with developmental coordination disorder (DCD) [19]. In a relatively large sample of school-aged children (*n* = 2119) in Hong Kong, PA was found negatively related to cardiovascular risk factors [20]. In addition, lower levels of PA were associated with higher levels of resting heart rate among a representative sample of 14,842 Hong Kong children [21]. Moreover, a RCT demonstrated that a six-week exercise program of strength training, aerobic exercise, and agility training significantly improved lean body mass and total bone mineral content of Hong Kong children [22].

Findings of the health benefits of PA on weight-related indicators among Hong Kong children were inconsistent. For example, one study found that PA was negatively correlated with the sum of skinfolds in boys although not in girls [23]. However, in another study, no association between PA and overweight status from Hong Kong primary school students aged 9–12 was found [24].

Psychological benefits of PA among Hong Kong children included perceived physical competence, better sleep, and higher quality of life. Based on a survey of 1012 Hong Kong children, PA was found positively related to children’s perceived physical competence [25]. Regarding child cancer survivors, PA was found to be negatively associated with cancer fatigue [26] and significantly predicted children’s quality of life [27]. Furthermore, a recent RCT intervention demonstrated that a 12-week PA intervention using basketball skill learning significantly improved sleep quality of Hong Kong children with ASD [28].

The relations between PA and children’s academic performance were inconsistent. For example, a study found that sport participation was positively related to academic performance for 11–12 year-old schoolchildren [29]. However, in another study, no significant association between PA and children’s academic performance was found [30].

#### 3.2.2. Patterns of PA

The levels of PA among Hong Kong children are relatively low (see Table 3). An observational study targeting Hong Kong children aged 6–8 on their activities at home and school found that only 18.14% of children’s time was active, while only 3.04% was very active [31]. In another study, only 9.1% of the boys and 11.9% of the girls reached the recommended levels of PA [32]. Particularly, obese children spent 30% less time in PA than non-obese children [33].

Different patterns of PA between boys and girls were revealed. It seems that boys spent more total and outside-school MVPA than girls spent, even though girls spent more time than boys did at in-school MVPA [34]. A three-day PA recall study found that boys attended more after-school sport-type activity lasting 30 min than girls [35].

Patterns of PA also showed differences between school PE environments and PA programs. For example, a recent study found that children were more active with active PE teachers than with less active PE teachers [36]. Of a related issue is that Hong Kong children participating in organized PA generally reported more times of after-school PA that lasts 30 min [37]. Moreover, interventions studies found that Hong Kong children spent more percentage of MVPA when at the interactive [38] and running game [39].

Hong Kong children with special educational needs spent much less time performing PA than healthy children. For example, a recent study found that 6.1% children with intellectual disabilities (ID) engaged in MVPA 60 min per day and 91.6% children with ID engaged in MVPA below 60 min per day. This was even worse for children with multiple disabilities (e.g., visual impairment, hearing impairment, physical disability, and ID) [40]. In the other two studies, it was found that children with multiple disabilities spent an average of four to six times of PA that lasted 10–30 min each month [41], and the averaged school-day MVPA of children with multiple disabilities was 18.6 min during the winter and 15.6 min during the summer [42].

#### 3.2.3. Measures of PA and Related Constructs

Regarding the measures of PA, both self-report scales and device (i.e., pedometer) for measuring PA were validated (see Table 4). For example, the Modified Physical Activity Questionnaire for Children (MPAQ-C) was examined among Hong Kong children and their parents, showing satisfactory internal consistency reliability, test-retest reliability, and criteria-related validity with pedometer-measured PA [43]. Likewise, the Chinese version of the Physical Activity Questionnaire for Older Children (PAQ-C) was tested in a sample of older children in Hong Kong, showing satisfactory internal consistency reliability, test-retest reliability, and criteria-related validity with accelerometers [44]. Moreover, the modified Chinese version Children’s Leisure Activities Study Survey (CLASS) showed acceptable test-retest reliability and criteria-related validity with accelerometer-measured PA [45]. Regarding device-measured PA, the Yamax Digiwalker DW-200 pedometer (Yamasa Tokei Keiki Co., Ltd., Tokyo, Japan) demonstrated satisfactory criteria-related validity with Children Activity Rating’s Scale among a group of young children [46]. An observational tool, the Behaviors of Eating and Activity for Children’s Health Evaluation System (BEACHES) showed high internal consistency and criteria-related validity with accelerometer-measured MVPA among five children with cerebral palsy [47].

Scales measuring the PA-related social environment have also been developed and validated among children in Hong Kong. For example, the PA-related Neighborhood Informal Social Control Scale for Parents of Preschoolers (PANISC-PP) was developed [48], with satisfactory factorial and construct validities and internal reliabilities further established [49]. Furthermore, parent-report measures of home and neighborhood environmental correlates of PA for preschool-aged children in Hong Kong was also developed with satisfactory internal consistency reliabilities and test-retest reliabilities [50].

PA-related psychological scales have been adapted and validated. For example, self-report measures of self-efficacy, enjoyment and social support for PA were adapted and validated in Hong Kong children with satisfactory internal consistency reliabilities, test-retest reliabilities, and criteria-related validities with self-reported PA [51]. In addition, a self-efficacy for PA measure was tested in Hong Kong children with satisfactory internal consistency reliability and factorial validity [52]. Lastly, a scale measuring child- and parent-reported psychosocial and environmental correlates of PA demonstrated acceptable internal consistency reliabilities and test-retest reliabilities [53].

#### 3.2.4. Correlates of PA

There were 18 studies on the correlates of PA (see Table 5). In line with the socio-ecological model [54,55], we classified the studies into five main aspects: (a) community-level correlates (e.g., built environments), (b) organizational correlates (e.g., school), (c) interpersonal correlates (e.g., family, friends, social network), (d) individual correlates (e.g., knowledge, attitudes, and skills), and (e) correlates from multiple levels.

Regarding the community-level correlates, studies focused on the influences of neighborhood built environment on PA among children. For example, one study found that children in the close-to-recreational-facility neighborhood had a higher level of accelerometer-measured MVPA than children in the far-to-recreational-facility neighborhood [56]. In addition, a qualitative study revealed 16 environmental factors that were most important to PA among children including facilitators (e.g., sufficient lighting, few cars on roads, and convenient transportation) and barriers (e.g., crimes nearby, too much noise, and too many people in recreation grounds) [57].

Organizational correlates of PA among children mainly relate to the school contexts in Hong Kong. In one study, the length of PE lesson was found negatively associated with children’s MVPA percentage during PE lessons, while lesson context, lesson content, temperature, and active teacher behavior significantly predicted children’s MVPA percentage during PE lessons [58]. Likewise, children’s percentage of MVPA was found positively related to the time teachers spent observing students, but negatively related to the time teachers spent instructing and managing [59]. Moreover, a study found that a change from passive to active travel to school was positively related to changes in the MVPA time [60].

In terms of the interpersonal correlates, parental influences on children’s PA were examined. In a study, parental influence was found having direct and indirect effects on children’s PA via children’s PA perception and physical self-perceptions [61]. Another study found that parents’ perceptions of children’s competence and exercise benefits predicted parental support, which in turn predicted children’s PA [62]. Nonetheless, a study found that parents’ encouraging on their children’s PA includes instrumental, motivational, and conditional support, while discouraging children’s PA includes parental safety concerns, focus on academic achievement, lack of time and resources, and promotion of sedentary behaviors [63].

Regarding individual correlates, studies examined children’s motor ability and fundamental movement skills (FMS) and psychological correlates. Children’s locomotor skills was significantly associated with perceived movement skill competence, which was significantly related to children’s subjective and objective PA [64]. In addition, motor ability was found positively correlated with the total PA among Hong Kong children with developmental coordination disorder (DCD) [65]. Likewise, locomotor skill and running of FMS proficiency were significantly related to PA among children with DCD [66]. In another study, the weekday and weekend PA of children with cerebral palsy were found significantly related to process-oriented and product-oriented FMS proficiency [67]. Furthermore, children’s PA self-efficacy and their autonomous motivation significantly and positively predicted their self-reported PA and accelerometer-assessed MVPA [68].

The correlates of children’s PA were also examined from multiple levels at the same time. In a study, father’s role modeling was found significantly predicted attraction to PA in overweight boys but not girls, while children’s perceived competence was found significantly predicted the attraction to children’s PA [69]. In another study, parental role modelling for PA and preference for outdoor play were found significantly associated with both questionnaire-based and accelerometer-assessed MVPA, with attractive natural sights significantly related to accelerometer-assessed MVPA and social network significantly associated with questionnaire-based MVPA [70]. Likewise, socio-demographic and family/home characteristics were found significantly correlated with parenting practices encouraging and discouraging PA, while parent-perceived neighborhood characteristics only significantly correlated with parenting practices discouraging PA [71]. Moreover, self-efficacy and school sport teams were found significantly associated with MVPA for boys, while school sport teams, homework, peer support for PA, and home PA environment were significantly associated with PA for girls [72]. In another study, physical condition, misunderstanding about PA, emotional disturbances, and social influences were found having negative impacts on PA among children with cancer [73].

#### 3.2.5. Interventions for Promoting PA

There were 10 studies focusing on promoting PA among Hong Kong children, including healthy children (*k* = 4), children with disabilities (*k* = 3), and children with cancer (*k* = 3) (see Table 6). There are various approaches used to promote PA among healthy children. For example, a modified “Play & Grow” program for Hong Kong children aged 2–4 years old and their parents significantly improved the time spent on PA by children’s mothers although the intervention did not lead to significant changes in children’s PA [74]. A recent RCT intervention on promoting diet and PA of children aged 8–12 using health video game showed that self-reported PA significantly increased, although the effect cannot be maintained after 8–10 weeks [75]. In addition, a pre-post quasi-experimental design of motivational interview was found effective on significant improvement in PA among obese children aged 9–11 [76]. Moreover, a heart-rate feedback intervention was found effective on short-term change of PA in Hong Kong children, including total daily PA and vigorous PA but not moderate PA [77].

There is a growing number of interventions aimed to promote PA among Hong Kong children with disabilities since 2010. In one study, FMS training did not lead to significant changes in weekday PA for children with and without cerebral palsy, but significantly increased weekend MVPA for children with cerebral palsy [78]. In another study, a school-based FMS training program resulted in increased short-term and long-term PA change in children with and without developmental coordination disorder (DCD) [79]. Furthermore, a FMS training showed significant interaction effects on PA volume and light PA but no significant main and interaction effects for MVPA among Hong Kong children with DCD [80].

Promoting PA for children with cancer is a key area in Hong Kong. A RCT study of integrated adventure-based training and a health education program demonstrated effectiveness in promoting regular PA among childhood cancer survivors [81]. Likewise, a RCT of a four-day adventure-based training was demonstrated effective in promoting PA among childhood cancer survivors with significantly improved PA levels of the experimental group than the control group at 6-months and 12-months follow-ups [82]. Another RCT study demonstrated that integrated experiential training program are effective in promoting PA among children with cancer, in which experimental group showed significantly higher levels of PA than control group at 6 months and 9 months after the start of the intervention [83].

## 4. Discussion

Following the brief description of the findings on each of the 63 eligible studies within the results section, we will further discuss and evaluate the general patters of the findings in the discussion section. Specifically, we discuss the characteristics of the studies, as well as the five main categories: health benefits of PA, patterns of PA, measures of PA and related constructs, correlates of PA, and interventions for promoting PA.

Overall, there is a growing number of studies published after the year of 2011 as compared to previous years from 1997 to 2010. This is a positive trend with more studies focusing on the Hong Kong context in terms of PA among children. This may also indicate that researchers are starting to pay more attention to the importance of studying and promoting PA among the preschool and school-aged children in Hong Kong. In addition, majority of the studies focused on the school-aged children as compared to the studies focused on the preschoolers. Given that preschool years is a critical period to intervene, more PA studies should be conducted on this group of age range such as family-based PA studies [84]. Moreover, sample sizes of the studies in the current review are small to medium with 36.1% of studies reporting small sample size below 100 and 39.3% of studies had a medium sample size between 101 and 500. To obtain sufficient power and reach conclusions that are generalizable, future studies should recruit a relatively large and representative sample. Regarding the PA measures, the majority of studies used self-reported scales while one-fourth of studies used accelerometers/pedometers or both accelerometers and self-reported scales. Researchers should collect data from both the device-measured PA using accelerometers and self-reported PA, given the fact that both types of measures are not synonymous and both have value [85]. Lastly, majority of the studies adopted a cross-sectional design, with eight studies using a longitudinal design, and 10 studies applying the RCT design. To move forward, more RCT studies are required given the limitation of causal inference inherent in cross-sectional and longitudinal designs [86].

The health benefits of PA on Hong Kong children are consistent with previous international literatures. These benefits include skeletal mass [87], hear-rate monitoring [88], cardiovascular risk factors [89], sleep quality [90], quality of life [91], and perceived competence [92]. Yet, we found some discrepancies with the international literature in terms of PA and academic performance [93] and weight-related markers [94]. A potential possibility for the mixed findings on weight-related indicators is that the intake of energy-dense foods and beverages was not taken into consideration along with PA [95]. Regarding the mixed findings on PA and academic performance, it might because the cross-sectional design with future studies needed to adopt longitudinal or experimental designs to confirm the causal relationships from PA to academic performance. Another potential explanation is that there might be cultural differences in Hong Kong that the academic performance is highly valued as compared to PA [29], so it is difficult to find the PA-academic performance relation. Furthermore, the inconsistencies might arise from distinct operational definitions and respective methodological decisions. For example, the weight indicators using skinfold and BMI represent fundamentally different views and procedures. Likewise, conceptual and methodological variations exist on how to measure academic performance. For instance, the measure of perceived academic performance and objective assessment results like the GPA might lead to substantial differences. In addition to the measures of PA and the other sports-specific indicators, important considerations on the conceptualization and operationalization of the related measurements should be raised. For future research, there is a growing interest on the benefits of PA on the mental health issues [96] and cognitive function [97]. As such, future PA research in Hong Kong can explore the health benefits of PA on mental health, cognitive function, and academic performance of Hong Kong children [98].

Generally, there is a low level of PA among children in Hong Kong as reported in the studies of this review. This is in line with the findings of Hong Kong’s 2018 report card on physical activity for children and youth [4]. There seems to be different patterns of PA among Hong Kong children between boys and [34,35]. Future research in Hong Kong should consider focusing on promoting PA among young girls in particular [99]. Focusing on cultivating active PE teachers [36], promoting after-school organized PA programs [37], and adopting interactive and running activities for promoting PA via active video games [38,39] are also recommended. Overall, children with special educational needs spent much less time on PA [40,41,42]. In order to develop a more inclusive society in Hong Kong [5], it is important to promote disability-appropriate PA among children with special educational needs [100]. Researchers, stakeholders of kindergartens and primary schools, and policy makers in Hong Kong are recommended to continuously focus on reducing barriers as well as promoting the facilitators from the personal, social, environmental, and policy levels in order to increase PA among children with special educational needs [101].

PA measures have been adapted and validated for use in Hong Kong children and their parents. In this scoping review, we focused on both the measures of PA and PA-related constructs. The PA measures [43,44,45,46] provided valid tools for collecting self-report data on Hong Kong children’s PA for both correlational and interventional studies. Validating the observational tool for measuring PA among Hong Kong children [47], the PA-related home and neighborhood measures [48,49,50] are also very important which provided various approaches for collecting different types of PA data. The measures of PA-related psychological [51,52,53] can be used for exploring the psychological benefits and the potential changing mechanisms of PA interventions. Instead of solely assessing the PA self-efficacy, PA enjoyment, and PA social support, the theory informed PA-related psychological measures can also be used to examine the changing mechanisms of psychosocial theory-based PA interventions for promoting PA among children and their parents, such as the intention formation, planning, and habits [102].

Research on PA correlates were in line with the social ecological model [54,55]. The community correlates of PA [56,57] can inform policymaking and the development of PA interventions, such as providing recreational facilities in the neighborhood, sufficient lighting, and building bridges or tunnels. For the organizational correlates, reduced PE lesson length and active PE teachers’ behaviors [58] and PE teachers observing [59] related to higher levels of children’s MVPA. Future PE teachers-led and school-based PA promotion programs should consider training PE teachers to be mindful of the contexts and contents of their PE lessons but also their own behaviors. In terms of the interpersonal correlates, evidence provided support for parental influence and parental support on the physical activity of children [61,62]. Further studies should treat the entire family system as a source of interpersonal correlates on PA of children by considering the relationships linking family and PA among children, including the family structure/arrangements, family, and family members as stakeholders [103]. In this review, correlates of children’s PA were examined from multiple levels [70,71,72,73], demonstrating that Hong Kong children’s PA is influenced by more than one type of socioecological correlate [72]. Future studies should continue to examine multiple levels of PA correlates in order to get an overall picture of the relative contribution of various socioecological influences.

There were different approaches for promoting PA among healthy children, including educational sessions of parental PA practices [74], the use of video games embedded with story immersion [75], motivational interview plus telephone consultation [76], and educational session plus heart rate feedback [77]. FMS training was useful in promoting PA among Hong Kong children with cerebral palsy [78] and DCD [79,80]. Adventure-based training plus education [81,82] and integrated experiential training plus home visit [83] promoted PA among children with cancer. Findings of the current review echoed the call from a previous review [16] that we need more RCT studies for promoting PA among children in Hong Kong with effectiveness. In addition, there is a lack of using theories to explain the mediators of behavior change which are important and effective for promoting children’s PA [104]. Moreover, no intervention studies reported upon behavior change techniques (BCTs) in order to determine the most effective techniques in promoting children’s PA [105], and future interventions on promoting PA among children in Hong Kong should include the BCTs. Future studies in Hong Kong should also consider using complex interventions that contains several interacting components [106] to promote PA among children in Hong Kong. Complex interventions have been widely applied in public health practices, which can lead to great improvements in PA [107]. Researchers, practitioners, and policymakers should also consider implementing multicomponent interventions to promote PA among children in Hong Kong, such as school-based interventions with the involvement of family and community components.

### Limitations and Future Directions

Limitations of the current study should be acknowledged. First, we classified the studies into five main categories to describe and evaluate the studies. However, some variables are classified into more than one category. For example, we may classify health benefits of physical activity into the correlates of physical activity at the same time that determinants of PA can also be a consequence of PA participation. Future studies should adopt experimental designs to reveal the reciprocal relations between key variables and PA. Second, we determined the classification of eligible studies based on the main purposes of studies, yet many studies tested more than one aspect. For example, authors reported both the patterns of PA and the relationships of PA with correlates. Normally, we classified these studies as correlates. Yet, we can also use the patterns of PA information from the PA correlates studies to inform future studies try to change children’s PA patterns. Third, we used the inclusion criteria that studies will be included with the mean age of participants fall within the age range of 3–12 years old. This way, participants of some studies are children over 12 years old or below 3 years old. This is a compromised solution aiming to include more eligible studies focused on children, but it may also lead to biases. Future review should consider using a more strict inclusion criteria than the mean age range should fall within 3–12 years old. Fourth, different types of PA such as PE lessons, active travel, and sport participation were reviewed in the same categories. There might be discrepancies on the intensities of different types of PA such as light PA, MVPA, and vigorous PA. Future study should take the intensity of PA into consideration by focusing on a certain type of PA in addition to reviewing the overall PA. Last, we did not conduct the quality assessment due to the nature of the study as a scoping review. We have broad research questions and a wide range of study designs. Future research should consider conducting quality assessment in studies with similar designs such as interventions. Future research can also consider conducting a systematic review to provide an overall pattern of the direction of a certain effect. It will become more feasible to conduct a systematic review or meta-analysis to examine on the health benefits of PA or the effects of interventions to promote PA among children along with more and more research published promoting PA among children in Hong Kong.

## 5. Conclusions

This scoping review identified 63 studies on the PA of children in Hong Kong published from 1997 to 2020. Overall, Hong Kong children’s PA is generally low. In particular, girls and children with special educational needs had relatively lower levels of PA. The health benefits of PA included skeletal mass and cardiovascular capacity, quality of life, sleep quality and cognitive function, while we noted inconsistent findings for weight-related issues and academic performance. Based on the socio-ecological model, correlates of PA covered all different levels from community level, organizational level, interpersonal level, to individual level. With the measures of PA and related constructs ready for use, there is still a lack of high-quality interventions for promoting PA among Hong Kong children. Limitations of the literature include an over-use of cross-sectional designs, reliance on self-reported PA measures, and small to medium sample studies. On the promising side, there is a growing number of studies published in this field since 2011, and a growing trend for PA research focusing on children with special educational needs and diseases for developing a more inclusive Hong Kong society. We call for Hong Kong-based studies for promoting PA among preschool-aged children as well as studies with robust design, a large and representative sample, and a combination of self-report and device-assessed PA.

## Figures and Tables

**Table 1:** Study characteristics of physical activity (PA) research among children in Hong Kong.

Characteristics		Number of Studies	Percentages
Year of publication			
	2016–2020	25	39.7%
	2011–2015	20	31.7%
	2006–2010	11	17.5%
	1999–2005	7	11.1%
Age range of participants			
	Preschool children (3–5 years old)	9	14.3%
	School-aged children (6–12 years old)	51	81.0%
	Preschool and school-aged children (3–12 years old)	3	4.8%
Range of sample size ^a^			
	100 and below	22	36.1%
	101–500	24	39.3%
	501–1000	8	13.1%
	1000 and above	7	11.5%
Population			
	Healthy children	44	69.8%
	Children with special educational needs	13	20.6%
	Children with disease ^b^	6	9.6%
Types of special educational needs			
	Coordination disorder	5	38.5%
	Cerebral palsy	4	30.8%
	Multiple disabilities ^c^	2	15.4%
	Autism spectrum	1	7.7%
	Intellectual disorder	1	7.7%
Study design			
	Cross-sectional	40	63.5%
	Randomized controlled trial	10	15.9%
	Longitudinal	8	12.7%
	Qualitative	3	4.7%
	Non-randomized controlled trial	2	3.2%
Types of PA measures			
	Single type: Self-report	44	69.8%
	Single type: Device measured	11	17.5%
	Combined type: Self-report & devices	6	9.5%
	Single type: Objective observation	2	3.2%

Note: ^a^ Two observational studies did not report the sample size of children; ^b^ Cancer; ^c^ Multiple disabilities = visual impairments, hearing impairments, physical disabilities, intellectual disabilities, and social development problems.

**Table 2:** Health benefits of physical activity (PA) on children in Hong Kong.

Reference		Characteristics		Design	Health Benefits	Main Findings
		N	Age			
[19]	Tsang et al. (2012)	63	6–10	CS	Skeletal development	Limited PA was related to delayed skeletal development among pre-pubertal children with developmental coordination disorder (*r* = 0.339, *p* < 0.05).
[20]	Kong et al. (2010)	2119	6–20	CS	Reduced cardiovascular risk factors	PA was negatively related to Chinese youth’s cardiovascular risk factors (*r* = −0.455, *p* = 0.006) after adjusting for sex and pubertal stage.
[21]	Kwok et al. (2013)	14,842	6–18	CS	Resting heart rate	Higher levels of PA was associated with lower levels of resting heart rate (boys: *β* = −0.13, *p* < 0.001; girls: *β* = −0.10, *p* < 0.001).
[22]	Yu et al. (2005)	82	9–12	RCT	Lean body mass, Total bone mineral content	Obese children’s lean mass and total bone mineral content significantly increased after a six-week exercise program (strength training, aerobic exercise, and agility training).
[23]	Rowlands et al. (2002)	50	8–11	CS	Less body fatness	Objectively measured PA was significantly correlated with sum of skinfolds in boys (*r* = −0.50, *p* < 0.05) but not girls.
[24]	Wang et al. (2017)	894	9–12	CS	Prevalence of overweight	PA was not significantly related to prevalence of overweight in Chinese children (AOR = 0.95, 95%CI = 0.73, 1.23; *p* > 0.05).
[25]	Cheung & Mak (2014)	1012	9–14	CS	Perceived physical competence	PA was significantly related to perceived physical competence (*r* = 0.354, *p* < 0.001).
[26]	Ho et al. (2019)	400	7–18	CS	Less cancer fatigue	PA significantly related to fatigue among children surviving cancer (*r* = −0.56, *p* < 0.01).
[27]	Lam et al. (2016)	76	9–18	CS	Quality of life	PA predicted life quality among young cancer patients in Hong Kong (*β* = 0.72, *p* < 0.001).
[28]	Tse et al. (2019)	40	8–12	RCT	Sleep quality, Executive functions	After 12-week PA intervention of basketball skill learning, children with ASD’s sleep quality and inhibitory control significantly improved, but not working memory capacity.
[29]	Lindner (1999)	4690	9–18	CS	Academic performance	Sport participation significantly related to academic performance of children aged 11–12 (*r* = 0.12, *p* < 0.05).
[30]	Yu et al. (2006)	333	8–12	CS	Academic performance	PA was not significantly related to academic performance (*r* = −0.067, *p* > 0.05).

Note. CS = cross-sectional study; RCT = Randomized controlled trial; AOR = adjusted odds ratio; ASD = autism spectrum disorder; FMS = Fundamental movement skills.

**Table 3:** Patterns of physical activity (PA) among children in Hong Kong.

Reference		Characteristics		Design	PA Measures	Patterns of PA
		N	Age			
[31]	Johns & Ha (1999)	40	6–8	LS	Children’s health evaluation system	Percentage of active time spent at home and school: Active = 18.14%; Very active = 3.04%
[32]	Huang & Wang (2015)	1013	9–13	CS	Children’s Leisure Activities Study Survey	Percentage of children reached recommended PA, and mean MVPA per day: Boys = 9.1%, 178 min, Girl = 11.9%, 165 min.
[33]	Yu et al. (2002)	36	7–17	CS	Diary	Obese children spent 30% less time in physical activity than non-obese children.
[34]	Lam et al. (2010)	1147	9–13	CS	China Health and Nutrition Survey	Outside-school MVPA per week: Boys = 229 min, Girls = 212 min; In-school MVAP per week: Boys = 160 min, Girls = 163 min; Total MVPA per week: Boys = 389 min, Girls = 375 min.
[35]	Cheung (2017)	663	6–13	CS	Three-day physical activity recall	Times of after-school 30-min sports-type activity: Boys = 1.1; Girls = 0.7; Mother’s education/income: High = 0.72/0.75; Medium = 1.04/0.83; Low = 1.23/0.99. Father’s education/income: High = 0.71/0.71; Medium = 1.04/0.74; Low = 1.17/1.00.
[36]	Cheng (2020)	248	5–6	CC	Step count pedometer	Step counts during 30 min PE lesson with active/less active teacher: Boys = 1795/1010 steps; Girls = 1608/889 steps.
[37]	Cheung (2012)	456	10–12	CS	Three-day physical activity recall	After-school 30-min PA participation with/without organized PA: Light intensity PA: 5.55/6.28 times; Moderate intensity PA: 1.24/1.18 times; Hard intensity PA: 0.79/0.42 times; Very hard intensity PA: 0.25/0.05 times.
[38]	Sit et al. (2010) JPAH	70	9–12	LS	System for Observing Fitness Instruction Time	Percentage and averaged MVPA during the 60-min games: Bowling game = 38.9%, 22.4 min; Running game = 52.7%; 29.1 min.
[39]	Sit et al. (2010) IJP	60	9–12	LS	System for Observing Fitness Instruction Time	MVPA during game modes per time: Interactive bowling = 78 min; Computer bowling = 3.7 min; Interactive running game = 98.4 min; Computer running game = 4.3 min.
[40]	Wang et al. (2018)	524	8–16	CS	Global Physical Activity Questionnaire	Disabilities = ID. 6.1% children engaged in MVPA 60 min per day, and 91.6% children engaged in MVPA below 60 min per day.
[41]	Sit et al. (2002)	237	9–19	CS	Sport participation questionnaire	Disabilities = PD, VI, HI, MD, maladjustment. Averaged PA: Frequency = 4–6 times per month, Duration = 10–30 min per time.
[42]	Sit et al. (2019)	270	7–18	LS	ActiGraph accelerometer	Disabilities = VI, HI, PD, ID, and SD. Percentage and averaged MVPA during school day: Winter = 4.5%, 18.6 min, Summer= 4%, 15.6 min.

Note. MPA = Moderate PA; MVPA = Moderate-to-vigorous PA. VI = Visual impairment, HI = Hearing impairment, PD = Physical disability, SD = Social development, ID = Intellectual disabilities; DCD = Developmental coordination disorder; CP = Cerebral palsy, MD = Mental disability. CC = Case-control; CS = Cross-sectional study; LS= Longitudinal study.

**Table 4:** Measures of physical activity (PA) and related constructs among children in Hong Kong.

Reference		Characteristics		Measures	Internal Consistency Reliability	Test-Retest Reliability	Criteria-Related Validity
		N	Age				
[43]	Leung et al. (2016)	40	6–9	Modified Physical Activity Questionnaire for Children (MPAQ-C)	*α* = 0.79	ICC = 0.94; (*n* = 32)	Pedometers: *r* = 0.63
[44]	Wang et al. (2016)	742	8–13	Physical Activity Questionnaire for Older Children (PAQ-C)	*α* = 0.79	ICC = 0.82; (*n* = 94)	Accelerometer MVPA: *r* = 0.33
[45]	Huang et al. (2009)	220	9–12	Modified Chinese version of the Children’s Leisure Activities Study Survey (CLASS)	-	ICC = 0.71; (*n* = 139)	Accelerometer MVPA: Boy: *r* = 0.27, Girl: *r* = 0.48
[46]	Louie & Chan (2003)	148	3.3–5.1	Yamax Digiwalker DW-200 pedometers	-	-	Children Activity Rating’s Scale: *r* = 0.64
[47]	Sit et al. (2013)	5	7–13	Behaviors of Eating and Activity for Children’s Health Evaluation System (BEACHES)	-	-	Children with CP: Accelerometer and active category (ICC = 0.85).
[48]	Suen et al. (2014)	61 ^a^	3–5	PA-related neighborhood informal social control scale for parents of preschoolers (PANISC-PP) ^b^	T1: *α* = 0.74–0.90 T2: *α* = 0.78–0.90	ICC = 0.61–0.75;	-
[49]	Cerin et al. (2017)	394 ^a^	3–5	PA-related neighborhood informal social control scale for parents of preschoolers (PANISC-PP) ^b^	*α* = 0.82–0.89	-	-
[50]	Suen et al. (2015)	61	3–5	Measures of environmental correlates of physical activity for urban Chinese preschool-aged children ^c^	T1: *α* = 0.67–0.90 T2: *α* = 0.76–0.91	ICC = 0.45–0.93	-
[51]	Liang et al. (2014)	273	8–12	PA Self-efficacy (PASE); Adapted PA Enjoyment Scale (PAES); PA social support: social support from family (SSFA); social support from friends (SSFR).	PASE: *α* = 0.78 PAES: *α* = 0.90 SSFA: *α* = 0.86 SSFR: *α* = 0.90	PASE: ICC = 0.88 PAES: ICC = 0.82 SSFA: ICC = 0.86 SSFR: ICC = 0.91	Self-reported PA: PASE: *r* = 0.40, PAES: *r* = 0.23, SSFA: *r* = 0.40, SSFR: *r* = 0.35
[52]	Wang et al. (2017)	763	8–13	Self-efficacy for physical activity (PASE) ^d^	*α* = 0.91	-	-
[53]	Huang et al. (2011)	303	9–14	Psychosocial and environmental correlates measures of PA and screen-based behaviors ^d^	*α* = 0.50–0.75	ICC = 0.78–0.89	MVPA: Self-efficacy (*r* = 0.25), home PA environment (*r* = 0.14) and peer support for PA (*r* = 0.25).

*Notes*: α = Cronbach’s α; ICC = Intra Class-correlation Coefficients; PA = Physical Activity; MVPA = Moderate-to-Vigorous PA; CP = cerebral palsy; T1 = Time 1; T2 = Time 2. ^a^ parent-child dyads; ^b^ The subscales: (a) P = Personal involvement and general informal supervision; (b) C = Civic engagement for creation of better neighborhood environment; (c) E = Educating and assisting neighborhood children. ^c^ There are eight measures: Community cohesion (seven items), Perceived signs of physical and social disorder (17 items), Perceived risk of unintentional injury (five items), Perceived traffic safety and pedestrian infrastructure (eight items), Perceived stranger danger (four items), Availability of active-play equipment (eight items), Availability of passive-play equipment (seven items), and Places for child’s physical activity (11 items). ^d^ Both classical test theory (CTT) and item response modeling (IRM) were used. ^d^ Five subscales: Self-efficacy, Home PA environment, Peer support for PA, Family support for PA, and parental role modeling of TV viewing.

**Table 5:** Correlates of physical activity among Chinese children in Hong Kong.

Reference		Characteristics		Design	Correlates	Main Findings
		N	Age			
Community-level correlates						
[56]	He et al. (2015)	81	7–11	LS	Neighborhood environment	Children in the close-to-recreational-facility neighborhood had a higher level of accelerometer-measured MVPA as compared to children in the far-to-recreational-facility neighborhood (*p* < 0.05).
[57]	He et al. (2014)	34	10–11	QS	Neighborhood environment	16 environmental factors perceived as most important to children’s PA, including facilitators (e.g., sufficient lighting, bridge or tunnel, few cars on roads, and convenient transportation), and barriers (e.g., crimes nearby, too much noise, and too many people in recreation grounds).
Organizational correlates						
[58]	Chow et al. (2008)	105 ^a^	9–12	LS	Environmental; Instructor-related characteristics	Lesson context (*β* = 0.29), lesson content (*β* = 0.23), temperature (*β* = 0.20), and active teacher behavior (*β* = 0.25) significantly predicted children’s MVPA percentage during PE lessons.
[59]	Chow et al. (2015)	25 ^a^	3–6	CS	Teachers’ behavior during structured PE lessons	Proportion of lesson time teachers spent instructing (*r* = −0.21) and managing (*r* = −0.26) negatively related to children’s PA. Proportion of time teachers spent observing students positively related to children’s PA (*r* = 0.29).
[60]	Huang et al. (2017)	677	7–10	LS	School travel modes	A change from passive to active travel to school was positively associated with changes in the percentage of time spent in MVPA (*β* = 1.76).
Interpersonal correlates						
[61]	Cheung & Chow (2010)	872	10–13	CS	Parental influence	Parental influence imposed a total (*β* = 0.31) effect on children’s PA, which is divided as direct (*β* = 0.19) and indirect effect via children’s PA perception and physical self-perceptions (*β* = 0.12).
[62]	Leung et al. (2017)	478 ^b^	6–9	CS	Parental support; parents’ perceived competence & exercise benefits of children	Parents’ perceived children’s competence (*β* = 0.18) and exercise benefits (*β* = 0.29) predicted parental support, which in turn predicted children’s PA (*β* = 0.68).
[63]	Suen et al. (2015)	45	3–5	QS	Parental provision	Parental provision of instrumental, motivational, and conditional support can encourage child’s PA. Parental safety concerns, focusing on academic achievement, lack of time and resources, promotion of sedentary behaviors could discourage child’s PA.
Individual correlates						
[64]	Chan et al. (2019)	763	7–11	CS	FMS; Locomotor skills	Locomotor skills significantly related to perceived movement skill competence (*β* = 0.11), and perceived movement skill competence significantly related to objective PA (*β* = 0.59). Locomotor skills related to self-reported PA via perceived physical competence and enjoyment (*β* = 0.08).
[65]	Fong et al. (2011)	81	3–16	CS	Motor ability	Motor ability was positively associated with PA among children with developmental coordination disorder in Hong Kong (*r* = 0.264).
[66]	Yu et al. (2016)	130	7–10	CS	FMS proficiency	FMS proficiency was positively related to PA in Hong Kong children with respect to locomotor skill (*r* = 0.21) and running (*r* = 0.26).
[67]	Capio et al. (2012)	62	4–10	CS	FMS proficiency	Weekdays PA was significantly and positively related to process-oriented (*β* = 0.406–0.717) and product-oriented (*β* = 0.333–0.556) FMS proficiency among children with cerebral palsy. Similar patterns revealed for weekend PA and FMS proficiency.
[68]	Wang et al. (2016)	449	8–13	CS	Self-efficacy; autonomous motivation	Self-efficacy (*r* = 0.63) and autonomous (*r* = 0.50) motivation were positively associated with PA.
Correlates from multiple levels						
[69]	Lau et al. (2007)	104	8–12	CS	Parental influence; Child’s perceived competence	Father’s role modeling significantly predicted attraction to PA in overweight boys (*β* = 0.46) but not girls. Child’s perceived competence significantly predicted the attraction to PA by both boys (*β* = 0.63) and girls (*β* = 0.66).
[70]	Wong et al. (2016)	1265	8–12	CS	Home and neighborhood environments	Parental role modelling for physical activity (*β* = 0.046 and *β* = 0.146) and preference for outdoor play (*β* = −0.059 and *β* = −0.11) significantly related to objective and subjective MVPA. Attractive natural sights significantly related to objective MVPA (*β* = 0.101), social network significantly related to subjective MVPA (*β* = 0.095).
[71]	Suen et al. (2019)	411 ^b^	3–5	CS	Socio-demographic, family/home characteristics, neighborhood environments	Socio-demographic and family/home characteristics significantly related to parenting practices encouraging and discouraging PA. Parent-perceived neighborhood characteristics significantly related to parenting practices discouraging PA only.
[72]	Huang et al. (2013)	303	8–15	CS	Neighborhood environment; school sports teams; family and peer support; self-efficacy	After adjusting age and other significant correlates, self-efficacy (B = 0.89), school sport teams (B = 1.77) significantly associated with MVPA for boys. School sport teams (B = 1.50), homework (B = 0.19), peer support for PA (B = 0.95), and home PA environment (B = 1.21) significantly associated with PA for girls.
[73]	Lam et al. (2016)	25	9–18	QS	Factors contributing to low PA levels for Chinese children with cancer	Qualitative findings revealed that physical condition, misunderstanding about physical activity, emotional disturbances, and social influences had negative impacts on PA among children hospitalized with cancer.

*Notes*: GIS = Geographic Information System; PA = physical activity; SES = socioeconomic status; SB = sedentary behaviors; ST = sedentary time; CS = cross-sectional study; LS = longitudinal study; QS = qualitative study; FMS = fundamental movement skills; SES = socioeconomic status; MVPA = moderate-to-vigorous physical activities; LPA = light-intensity physical activities. The coefficients in the table are significant unless noted as insignificant. ^a^ physical education teachers of children; ^b^ parent of children.

**Table 6:** Interventions for promoting physical activity (PA) among children in Hong Kong.

Reference		Characteristics		Design	Interventions					
		N	Age		IG and CG	Weeks	Treatment	PA Measures	Time of Measures	Main Findings
[74]	Sobko et al. (2017)	240	2–4	One group	IG: Modified “Play&Grow” program.	12	45 min, once per week	IPAQ	Baseline and post-intervention.	No significant improvement on PA after intervention.
[75]	Wang et al. (2017)	179	8–12	QE-RCT	IG: Video game (Diab) CG: No intervention.	8–10	40 min, once per week.	PAQ-C; ActiGraph GT3X.	Baseline, post-intervention, and 8–10 week post-intervention.	Self-reported PA significantly increased after intervention (mean difference = 1.9, *p* < 0.05), but not maintained after 8–10 weeks.
[76]	Wong & Cheng (2013)	185	9–11	QE-RCT	IG1: MI + ; ^a^ IG2: MI; ^a^ CG: No intervention	14	30 min, six-section program	7 consecutive days self-record exercise log.	Baseline and post-intervention.	Both MI+ and MI improved PA. MI+ had more calorie consumed from PA than MI (F = 5.24, *p* = 0.02)
[77]	McManus et al. (2008)	210	9–11	CT	IG-E: Education + HR feedback IG-NE: HR feedback CG: No intervention.	4	2 weeks with heart rate feedback, and 2 weeks without.	HR; Children’s attraction to physical activity scale.	Baseline, during intervention, and 6 month post-intervention.	HR feedback increased total daily PA (24%, *p* < 0.001) and vigorous PA (0.6%, *p* < 0.05), but effects do not persist when feedback removed.
[78]	Capio et al. (2014)	50	3–10	RCT	TP & Disabilities: CP IG: FMS training CG: No intervention	4	45 min, once per week	Uni-axial accelerometers	7-day pre and post intervention.	Significant changes in weekday PA for both training groups. Weekend MVPA significantly increased for FMS training of children with CP.
[79]	Sit et al. (2019)	131	6–10	RCT	TP & Disabilities: DCD IG: FMS training CG: Conventional PE lessons	8	40 min, once per week	ActiGraph active monitor (GT3X).	Baseline, post-intervention, 1 week, 3&12 months post intervention.	FMS training improved %MVPA on weekdays in all time of measurements, and weekends in 3-months after intervention.
[80]	Yu et al. (2016)	84	7–10	RCT	TP & Disabilities: DCD IG: FMS training CG: No intervention	6	35 min, twice per week	ActiGraph active monitor (GT3X).	Baseline, post-intervention, & 6-weeks post intervention.	Significant interaction effects on PA volume (*p* = 0.043) and light PA (*p* = 0.026) but no significant main and interaction effects for MVPA.
[81]	Li et al. (2013)	71	9–16	RCT	Disease: Cancer IG: 4-day adventure-based training & health education. CG: Placebo.	4 days	40 min, per session in day 1–3; 90 min for day 4 session.	CUHK Physical Activity Rating for Children and Youth	Baseline, & 3, 6, 9 months after the beginning of intervention.	Experimental group showed significantly higher PA stages of change (*p* < 0.001) and PA (*p* < 0.001) than control group at all follow-ups.
[82]	Li et al. (2018)	222	9–16	RCT	Disease: Cancer IG: 4-day adventure-based training program CG: Placebo.	4 days	From 09:00 to 16:45 per day	CUHK Physical Activity Rating for Children and Youth	Baseline, 6 and 12 months after the intervention began.	Experimental group improved PA levels than control group at the 6-month (*p* < 0.001) and 12-month (*p* < 0.001) follow-ups.
[83]	Lam et al. (2018)	70	9–18	RCT	Disease: Cancer IG: Integrated experiential training + home visit. CG: Placebo.	24	60 min, once per week.	CUHK Physical Activity Rating for Children and Youth	Baseline, and 6 and 9 months after the beginning of intervention.	Experimental group improved PA levels than control group at the 6-month (*p* < 0.001) and 9-month (*p* < 0.001) after start of intervention.

Note. FMS = Fundamental movement skills; IPAQ = International Physical Activity Questionnaire; PAQ-C = Physical activity questionnaire for older children; IG = Intervention Group; CG = Control Group; HR = heart rate; TP = Typically developing; CP = Cerebral Palsy; DCD = Developmental Coordination Disorder; ASD = Autism Spectrum Disorder; QE-RCT = Quasi-experimental randomized Controlled Trial; RCT = Randomized Controlled Trial; CT = Controlled trial; CUHK = The Chinese University of Hong Kong. ^a^ MI = Motivation interviewing; MI + = Motivation interviewing with telephone consultation for parents.
